# Multidrug-resistant profile and prevalence of extended spectrum β-lactamase and carbapenemase production in fermentative Gram-negative bacilli recovered from patients and specimens referred to National Reference Laboratory, Addis Ababa, Ethiopia

**DOI:** 10.1371/journal.pone.0222911

**Published:** 2019-09-25

**Authors:** Degefu Beyene, Adane Bitew, Surafel Fantew, Amete Mihret, Martin Evans

**Affiliations:** 1 Ethiopian Public Health Institute, Clinical Bacteriology and Mycology Research Case Team, Addis Ababa, Ethiopia; 2 Department of Medical Laboratory Sciences, College of Health Sciences, Addis Ababa University, Addis Ababa, Ethiopia; 3 American Society for Microbiology, New York, New York, United States of America; CNRS: BIOM Integrative Biology of Marine Organisms, FRANCE

## Abstract

**Background:**

The emergence of multidrug-resistance (MDR), production of extended-spectrum β-lactamases, and carbapenemase in members of fermentative gram-negative bacilli are a serious threat to public health.

**Objective:**

The aim of this study was to determine the burden of multi-drug resistance, the production of extended-spectrum β-lactamases (ESBLs), and carbapenemase in fermentative Gram-negative bacilli in Ethiopian Public Health Institute.

**Materials and methods:**

A cross-sectional study was carried out from December 2017 to June 2018. Different clinical samples were collected, inoculated, and incubated according to standard protocols related to each sample. Bacterial identification was performed by using the VITEK^R^ 2 compact system using the GN^R^ card. Antimicrobial susceptibility testing was carried out by the Kirby-Bauer disc diffusion method. Production of ESBL and carbapenemase were confirmed by combination disc and modified Hodge Test method respectively.

**Results:**

A total of 238 fermentative Gram-negative bacilli were recovered during the study period, among which *E*.*coli* were the predominant isolates followed by *K*. *pneumoniae*. The highest percentage of antibiotic resistance was noted against ampicillin (100%) followed by trimethoprim/sulfamethoxazole (81.9%). The isolates showed better sensitivity towards carbapenem drugs. Out of 238 isolates, 94.5% were MDR and of which 8.8% and 0.8% were extensively and pan drug resistant, respectively. Nearly 67% and 2% of isolates were producers of ESBL and carbapenemase, respectively. The isolation rates of MDR, ESBL, and carbapenemase producing stains of the isolates were ≥70% in intensive care unit while the isolation rates in other wards were ≤25%.

**Conclusions:**

The findings of this study revealed that the burden of MDR and ESBL was high and carbapenemase producing isolates were also identified which is concerning. This situation warrants a consistent surveillance of antimicrobial resistance of fermentative Gram-negative bacilli and implementation of an efficient infection control program.

## Introduction

Fermentative Gram-negative bacilli (FGNB), belonging to the family of Enterobacteriaceae, are an important cause of diseases in humans, among which urinary tract infections, bloodstream infections, hospital- and healthcare-associated pneumonia, and a number of intra-abdominal infections are the most important [1, 2]

Antimicrobial resistance in this group of bacteria has been recognized by the World Health Organization as one of the most significant problems challenging human health [3]. This problem is further compounded by the emergence of multi-drug resistant (MDR), β-lactamase, and carbapenemase-producing bacterial pathogens [1, 4]. Acquisition and transferring of antibiotic resistance genes within or via different species of Gram-negative bacteria through mobile plasmids and transposons are reported to be the principal cause of the production of β-lactamases [5, 6]. Of particular importance is the production of extended-spectrum β- lactamases (ESBLs) that have the capacity to hydrolyze higher generation cephalosporin and cause resistance to many drugs including the third-generation cephalosporines, such as cefotaxime, ceftriaxone, and ceftazidime [7, 8]. Extended-spectrum β- lactamases producing FGNB have also been identified to coexist with resistance to other antimicrobial classes [9, 10] rendering the most useful drugs ineffective ultimately limiting treatment choices for infections. The main drivers for the development of resistance are antimicrobial selection pressure and the spread of the resistant organisms [11, 12]. Widespread and indiscriminate use of a broad-spectrum antimicrobial agent by a physician to treat an infection not only impacts the specific pathogen causing the disease but also kills populations of susceptible organisms that form a part of normal flora. In addition, the widespread use of antimicrobials as growth promoters in agriculture and animal husbandry creates a selective pressure that favours bacteria that are resistant to microbial, which can easily transferred to human through various chains [13, 14].

Although carbapenem antibiotics have been used as a last alternative to treat infections caused by multidrug-resistant FGNB. This is due to the fact that carbapenems are β-lactam drugs that are structurally different from penicillins and cephalosporins that have the widest spectrum of activity among the β- lactams with excellent activity against members of the FGNB. However, the activity of these antibiotics has been impaired by the development of drug-resistant strains against potent carbapenems due to extensive exposure of bacteria to antibacterial agents [15, 16].

The emergence and global spread of multidrug-resistance amongst bacterial pathogens implicated in causing both nosocomial and community-acquired infections are a major threat to public health everywhere. [17, 18]. The problem is far more important in bacterial species belonging to the FGNB because of their ubiquity in the environment and the relative ease of acquisition of plasmids containing genes that encode for ESBLs and other resistance genes that confer resistance to many other classes of antibiotics [7, 19, 20].

Despite the escalating burden of multidrug resistance (MDR), ESBLs and carbapenemase production in FGNB across the globe, data regarding the prevalence of MDR, ESBLs, and carbapenemase-producing FGNB in Ethiopia are limited. The objective of the present study is to determine the prevalence of MDR, ESBL and carbapenemase-producing FGNB. Their reliable determination plays a vital role in the successful management of infection and implementation of valid therapeutic strategies.

## Materials and methods

This prospective cross-sectional study was conducted at Ethiopian Public Health Institute (EPHI) in Clinical Bacteriology and Mycology Reference Laboratory from December 2017 to June 2018. The EPHI clinical microbiology laboratory is the only national reference and research laboratory where patients from different parts of the country are referred for culture ID and sensitivity tests. The laboratory is accredited by Ethiopian National Accreditation Office (ENAO) as a referral bacteriology laboratory since July 2017. The African Society for Laboratory Medicine (ASLM) awarded this laboratory with a certificate of recognition for achieving ISO accreditation and best practice in Laboratory Medicine after going through ASLM SLIPTA audits at their annual conference conducted in Abuja, Nigeria in 2018. Patients referred to Ethiopian Public Health Institute (EPHI) from Addis Ababa health facilities who are clinically suspected of bacterial infection and having request paper filled by physicians for culture and sensitivity and willingness to participate in the study were enrolled. Different clinical samples were submitted to the laboratory and processed following standard procedures. Specimens collected were inoculated onto appropriate isolation culture media (Blood culture broth, Blood agar, Chocolate agar, and MacConkey agar plate) and incubated at 35–37°C according to standard protocols for each sample. In cases where a delay in culturing was unavoidable, appropriate transport media were used. All commonly isolated fermentative gram-negative bacilli recovered from the various clinical specimen during the study period were included. Duplicate isolates from the same patient were excluded from the study. The data were collected using a pre-developed data collection form from the request paper. All the necessary variables were included in the request form and using data collection form these variables such as socio-demographic (age and sex), type of specimen, types of health facility from where the patient or specimen were referred, location of the patients at various wards, previous antibiotic exposure was collected by the principal investigator. Isolates were preliminarily characterized by colony characteristics and Gram-stain reaction. Bacterial identification was performed by the VITEK^R^ 2 compact system using the GN^R^ cards, in accordance with the manufacturer’s instructions (bioMérieux, France).

### Antimicrobial susceptibility testing

The antimicrobial susceptibility profile of isolates was determined by Kirby- Bauer disc diffusion method and the results were interpreted according to CLSI guidelines [21]. The following antibiotics were used; ceftriaxone(30μg), cefotaxime(30μg), ceftazidime(30μg), amoxicillin/clavulanic acid (20/10μg), amikacin (30μg), ampicillin (10μg), cefazolin (30μg), ciprofloxacin (5μg), gentamicin (10μg), nitrofurantoin (300μg), tetracycline (30μg), trimeth/sulfa (1.25/23.75μg), piperacillin (100μg), tobramycin (10μg), imipenem (10μg), meropenem (10μg), ertapenem (10μg), doripenem (10μg). The definition of Magiorakos et al [22] was used to classify bacterial isolates into MDR, extensively drug-resistant (XDR) and pan drug-resistant (PDR). MDR = resistant to at least one agent in three or more antimicrobial classes, XDR = Susceptible two or fewer antimicrobial classes, PDR = resistant to all antimicrobial agents in all antimicrobial classes.

### Test for ESBL production

All the strains which showed a diameter zone of inhibition of less than 27mm for cefotaxime and less than 22 mm for ceftazidime, were subjected to the ESBL confirmatory test. ESBL production was performed by a combination disc method in which discs of ceftazidime (CAZ) and cefotaxime (CTX) alone and in combination with clavulanic acid (CA) (10μg) were used [21]. The antibiotic discs were placed onto Mueller-Hinton agar plate seeded with a turbidity suspension of an isolate equal to that of a 0.5 McFarland turbidity standard. A difference of 5 mm between the zone of inhibition of a single disk and in combination with clavulanic acid was considered positive for an ESBL producer [21].

### Test for carbapenemase production

Bacterial isolates which were resistant to imipenem (IPM 10 μg), meropenem (MEM 10 μg) and ertapenem (ERTμ10) based on CLSI breakpoints [21] were subjected for confirmation for carbapenemase production. Confirmation for carbapenemase production in FGNB was conducted by Modified Hodge Test (MHT) where Mueller-Hinton agar plate was inoculated with a 1:10 dilution of a 0.5 densitometer standardized suspension of over-night sub-cultured *E*. *coli* ATCC 25922 and streaked for confluent growth using a swab. A 10 μg ertapenem disk was placed in the center, and each test isolate was streaked from the disk to the edge of the plate. A positive Modified Hodge Test (MHT) was indicated by clover leaf-like indentation of the *E*. *coli* ATCC 25922 growing along the test organism growth streak within the disk diffusion zone [21].

### Quality assurance

Performance of all media and antibiotics were checked by recognized standard strains using *E*. *coli* ATCC 25922, and *Pseudomonas aeruginosa* ATCC 27853. Standardization of carbapenemase and ESBL tests was performed using, *K*. *pneumoniae* ATCC BAA 1705 and ATCC 700603 and *E*. *coli* ATCC 25922 as positive and negative controls respectively.

### Data analysis and interpretation

The data was collected, cleaned and analyzed using SPSS version 20. Frequency and percentages of MDR, carbapenemase and ESBL producing gram-negative bacteria were calculated. Tables and figures were used for data presentation.

### Ethics and consent to participate

The study was carried out after the approval of the Internal Review Board (IRB) of Department of Medical Laboratory Sciences (DRERC/323/17/MLS) and permission letters were also obtained from Ethiopian Public Health Institute. Data collection was started after obtaining informed written consent from study subjects and assent the form was completed and signed by parents or guardians for those study subjects ≤ 16 years of age. All the information obtained from the study subjects were coded to maintain confidentiality.

## Results

Out of 947 different clinical samples were submitted to the laboratory during the specified time period, and bacterial pathogens were recovered from 306 among which 238 were fermentative gram-negative bacilli. Of these isolates, 138 were recovered from inpatients and 100 isolates were recovered from outpatients department. Among 238 isolates, 61.7% (147/238) were isolated from urine and 27.3% (65/238) were isolated from blood. *E*. *coli* was the dominant isolate accounting for 60.5% (144/238)) and *K*. *pneumoniae* was the second predominant species representing 30.3% (72/238) of the total isolates (Table 1). 136 (57.14%) of the isolate were recovered from patients that have one or two types of the previous history of antibiotic exposure before specimen collection. Their exposure includes the first-line antimicrobial agents up to the last treatment option (carbapenem). Of 136, 67 isolates were recovered from patients which were empirically treated with two types of different antibiotics before specimen collection whereas 69 of the isolates were recovered from patients which were empirically treated with one types of antibiotics. Of 69, 21 were isolated from patients empirically treated with CRO, followed by CIP (15), SXT (6) and of 67, 22 were isolated from patients empirically treated with CRO and VA, followed by Amp and Gn (17), CRO and Mer (6).

**Table 1:** Distribution of commonly isolated fermentative gram-negative bacilli among different specimen types.

	Urine Culture	Blood Culture	Pus Culture	Body Fluid Culture	Sputum Culture	Tracheal aspirate Culture	Ear Discharge Culture	Other specimen	Total
*E*. *coli*	117	18	3	2	2	0	1	1	144
*K*. *pneumoniae*	21	39	3	2	4	1	1	1	72
*Enterobacter*. *Cloacae*	6	5	2	0	1	2	0	0	16
*Citrobacter freundii*	3	3	0	0	0	0	0	0	6
Total	147	65	8	4	7	3	2	2	238

Percentage of the antibiotic resistance profile of bacterial isolates against 22 antibacterial agents is summarized in Table 2. The highest percentage of antibiotic resistance was noted against ampicillin (100%) followed by trimethoprim/sulfamethoxazole (81.9%), piperacillin (80.3%), and, tetracycline (80.3%). Fermentative Gram-negative bacteria showed the lowest resistance towards carbapenem drugs; imipenem, meropenem, doripenem, and ertapenem resistance rate is 1.7% for each. This was followed by amikacin and piperacillin-tazobactam combination with the overall resistance rate of 7.9% and 26.5%, respectively. (Table 2)

**Table 2:** Percentage of antibiotic resistance profile of commonly isolated FGNB against 22 antibacterial agents.

Types of Antibacterial agents		*E*. *coli* *N* (%)	*K*. *pneumoniae* *N* (%)	*E*. *cloacae* *N* (%)	*C*. *freundii* *N (*%)	Total %
Penicillins	Amp	144(100)	NT	16(100)	6(100)	100
	PIP	111(77.1)	65(90.3)	10(62.5)	5(83.3)	80.3
Beta-lactam and Beta-Lactamase Inhibitor	AMC	79(54.9)	51(70.8)	9(56.3)	4(66.7)	60.1
	PTZ	25(17.4)	16(22.2)	3(18.8)	0	26.5
Cephalosporin	KZ	107(74.3)	65(90.3)	13(81.3)	5(83.3)	79.8
	CRX	103(71.5)	60(83.3)	10(62.5)	5(83.3)	74.8
	CRO	100(69.4)	60(83.3)	10(62.5)	5(83.3)	73.5
	CTM	101(70.1)	60(83.3)	10(62.5)	5(83.3)	73.9
	CAZ	101(70.1)	60(83.3)	10(62.5)	5(83.3)	73.9
	CEF	101(70.1)	60(83.3)	9(56.3)	5(83.3)	73.5
Floroquinolones	CIP	92(63.9)	37(51.3)	5(31.3)	3(18.8)	57.6
	NOR	78(63.4)	13(52)	9(33.3)	2(66.7)	42.9
Carbapenems	IMP	0	4(5.6)	0	0	1.7
	MER	0	4(5.6)	0	0	1.7
	DOR	0	4(5.6)	0	0	1.7
	ERT	0	4(5.6)	0	0	1.7
Aminoglycosides	AMK	9(6.3)	9(12.5)	0	1(16.7)	7.9
	GN	55(38.2)	46(63.9)	6(37.5)	1(16.7)	45.4
	TOB	57(39.6)	51(70.1)	6(37.5)	3(50)	49.2
Tetracycline	TET	120(83.3)	57(79.2)	10(62.5)	4(66.7)	80.3
Sulfamethoxazole-trimethoprim	SXT	116(80.5)	64(88.9)	10(62.5)	5(83.3)	81.9
Nitrofurantoin,	F	85(59)	39(54.2)	7(43.8)	1(16.7)	55.5
Total		144	72	16	6	238

AMC-Amoxicillin-Calvulanic acid, AMK-Amikacin, Amp-Ampicillin, CAZ-Ceftazidime, CFP-Cefepime, CPR-Ciprofloxacin, CRO-Ceftriaxone, CRX-Cefuroxime, CTX-Cefotaxime, DOR-Doripenem, ERT-Ertapenem, F-Nitrofurinatoin, GN-Gentamycin, IMP-Imipenem, KZ-Cephazolin, MER-Meropenem, NOR-Norfloxacin, PIP-Piperacillin, PTZ-Piperacillin-Tazobactem, SXT- Trimethoprim -sulfamethoxazole, TET-Tetracycline, TOB-Tobramycin.NT-Not test, O—no resistance identified.

Out of 238 fermentative Gram-negative bacilli isolates, 94.5% were MDR of which 8.8% and 0.8% were XDR and PDR, respectively. Among 144 strains of *E*. *coli*, 99.3% were MDR of which 18.1% were XDR. Similarly out of 72 isolates of *K*. *pneumoniae*, 90.3% were MDR of which 11.2% and 2.8% were XDR and PDR respectively. 83.3% and 75% strains of *C*. *freundii* and *E*. *cloacae* were MDR but none of the strains of the two species were XDR and PDR producers. Nearly, 67% and 2% of FGNB were producers of ESBL and carbapenemase, respectively. Extended-spectrum β- lactamases producing were produced in 76.4%, 63.2%, 62.2% and 50% of *K*. *pneumoniae*, *E*. *coli*, *E*. *cloacae*, *C*. *freundii* respectively. None of the strains of *E*. *coli*, *E*. *cloacae*, and *C*.*freundii* produced carbapenemase but, carbapenemase was produced by 5.6% of *K*. *pneumoniae* strains (Table 3).

**Table 3:** Percentage prevalence of multidrug resistance, ESBL and carbapenemase production in commonly isolated fermentative Gram- negative bacilli.

Bacterial species	MDR (%)	XDR (%)	PDR (%)	ESBL (%)	Carbapenemase (%)
*E*. *coli (144)*	99.3	18.1	0	63.2	0
*K*. *pneumoniae (72)*	90.3	11.1	2.8	76.4	5.6
*E*. *cloacae (16)*	75	0	0	62.5	0
*C*. *freundii (6)*	83.3	0	0	50	0
Total(238)	94.5	8.8	0.8	66.8	1.7

MDR = resistant to at least one agent in three or more antimicrobial classes, XDR = Susceptible two or fewer antimicrobial classes, PDR = resistant to all antimicrobial agents in all antimicrobial classes, ESBL = Extended-spectrum β- lactamases producing.

**Fig 1:**
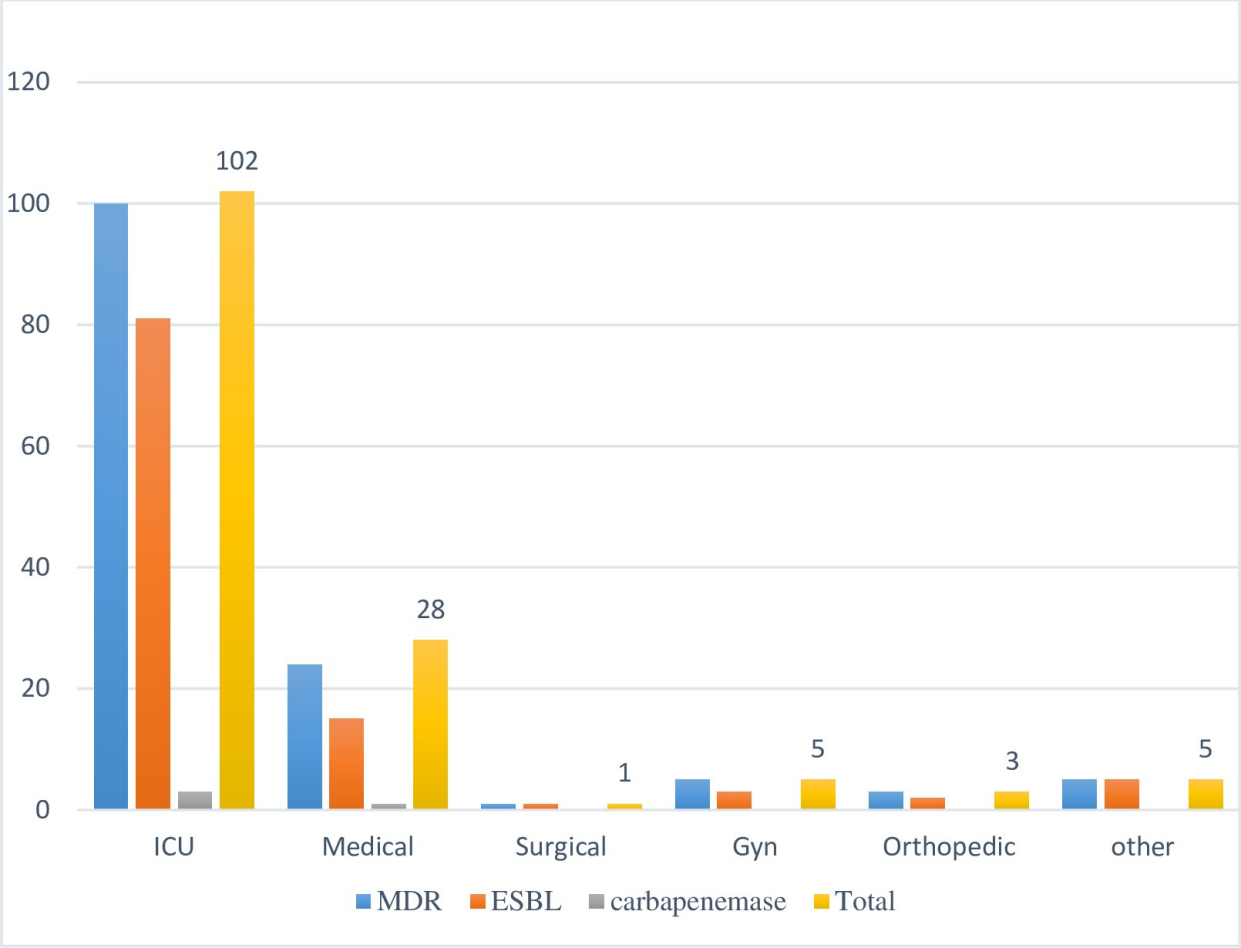
Prevalence of MDR, ESBL, and carbapenemase producing in FGNB among different wards.

Prevalence of MDR, ESBL and carbapenemase-producing FGNB by location of wards are depicted in “Fig 1”. Most of MDR resistant isolates were recovered from patients at intensive care unit ward (73%) followed by the medical ward (17.4%). Similarly, the isolation rate of ESBL producing isolates was higher among patients in the intensive care unit (75.7%) rather than from those in the medical ward. The recovery rate of carbapenemase-producing isolates were three-fold more among patients in an intensive care unit than in medical wards.

**Fig 2:**
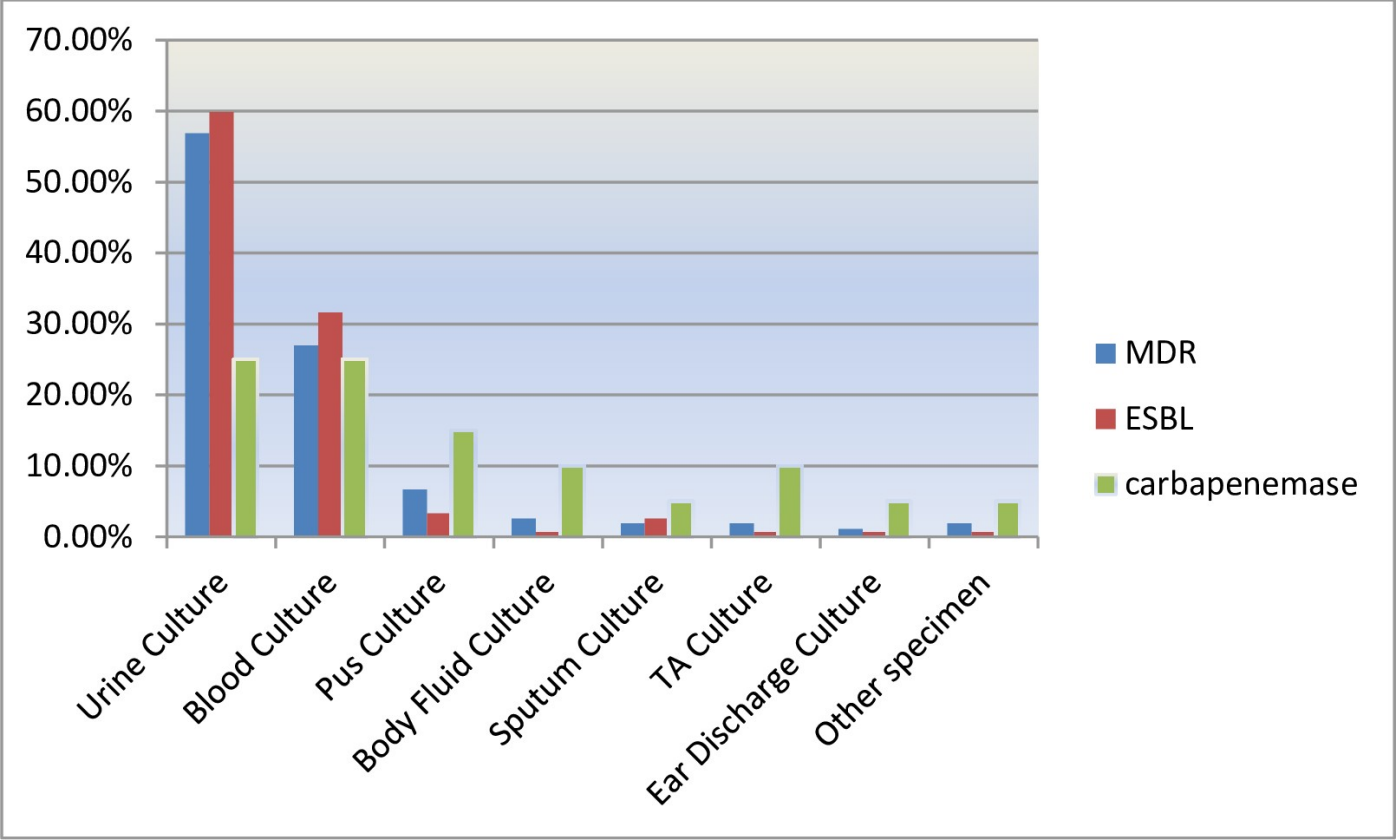
Prevalence of MDR, ESBL and carbapenamese producing FGNB among different clinical specimens.

The prevalence of MDR, ESBL and carbapenemase production in relation to clinical samples is presented in “Fig 2”. The prevalence of MDR isolates was greater in urine (62.5%) than blood (28.4%) specimens. The isolation rate of ESBL producing isolates was almost two-fold (58.5%) higher in urine than blood culture (33.3%). There was no difference in the recovery rate of carbapenemase-producing isolates between urine culture and blood culture.

**Fig 3:**
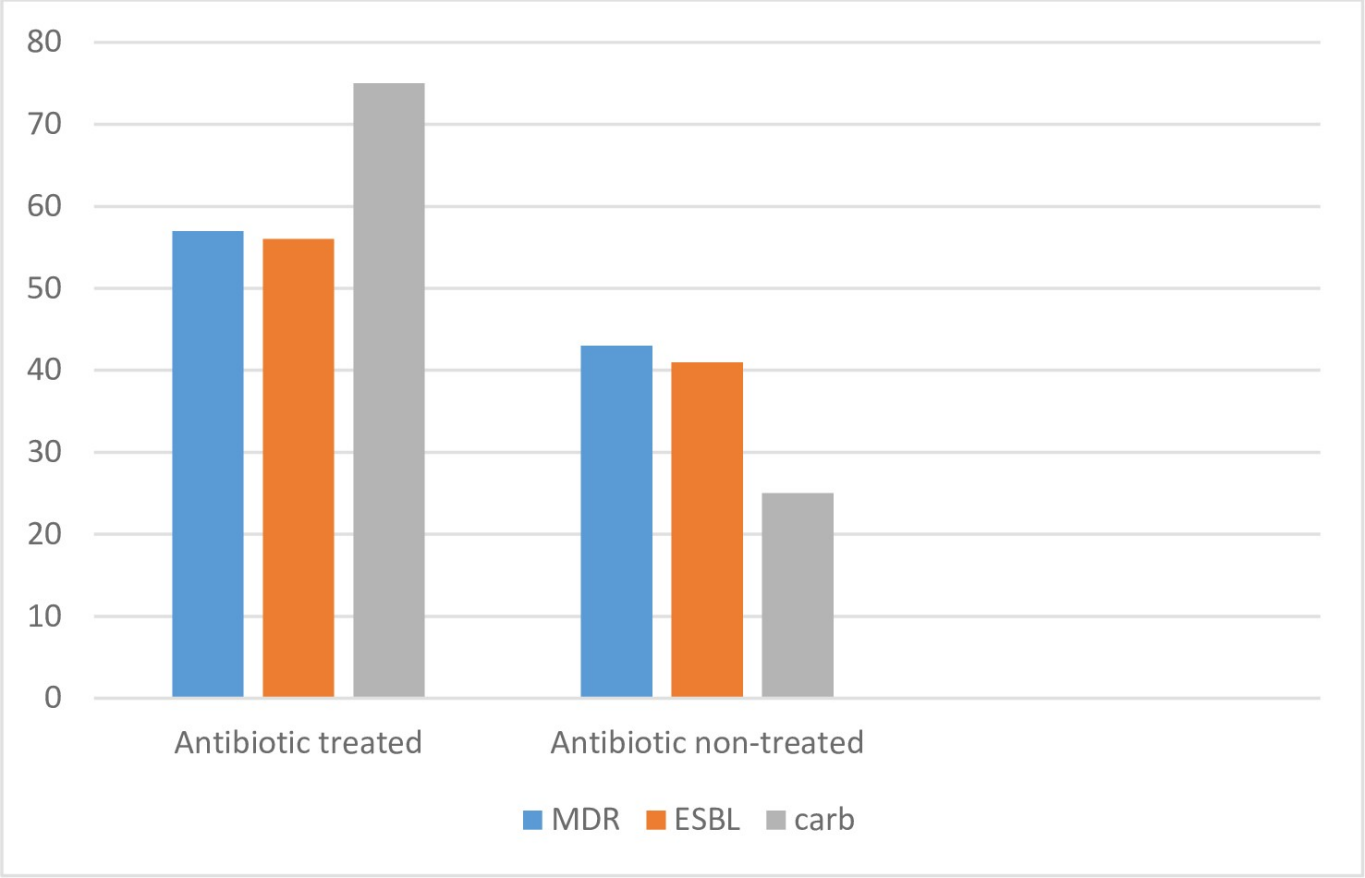
Prevalence of MDR, ESBL and carbapenamese producing FGNB between antibiotic exposed and non-exposed patients.

The prevalence of MDR, ESBL and carbapenemase-producing FGNB among antibiotic-treated and non-treated patients is shown in “Fig 3”. The prevalence of MDR, ESBL and carbapenemase production were higher among previously treated patients than non-treated. The ratio of MDR, ESBL and carbapenemase among treated to non-treated patients were 58. % to 41%, 58.8% to 41.2% and 70% to 30%, respectively.

## Discussion

In the present study, FGNB were tested against 22 antibacterial agents. Antibiotic resistance profile of FGNB against the first-line drugs were remarkably high. Resistance profile of FGNB against drugs of β-lactam/β-lactamase inhibitor combinations extends from 26.5% for piperacillin/tazobactam to 100% for ampicillin indicating that β-lactams with β-lactamase inhibitor demonstrate better activity. Antibiotic resistance profile of FGNB against other first-line drugs such as tetracycline, nitrofurantoin, and trimethoprim/sulfamethoxazole was also high. Again the overall resistance rate of FGNB against cephalosporin including the extended-spectrum β- lactam antibiotics was above 70%. Similarly, except for amikacin, the percentage antibiotic-resistance rates of FGNB against aminoglycosides and fluoroquinolone was above 40%. This finding correlates with the study conducted in other parts of Ethiopia Gondar University teaching hospital [23] and Nepal [24], where resistance proportion of the first line and other antibiotics were also high. However our finding disagrees with a study conducted at Dessie, Ethiopia [25], and Turkey, institute of Cardiology [26] where the lowest resistance proportion against the first line and other higher generation antibiotics were indicated. This variation in antibiotic resistance proportion might be due to geographical difference and study period.

Antibiotic resistance profile of FGNB against carbapenems, however, was seen to be low. Our finding in this regard was in line with earlier reports [23, 27]. Our study demonstrated that *E*. *coli*, *E*. *cloacae*, and *C*. *freundii* were 100% susceptible to all carbapenems tested, but 5.6% of the strains of *K*. *pneumoniae* were resistant to all the carbapenems antibiotics (doripenem, ertapenem, imipenem, and meropenem).

In the present study, out of 238 FGNB, 94.5% were MDR, among which 8.8% were XDR and 0.8% were PDR. There are published data on MDR fermentative gram-negative bacilli in Ethiopia but there are few data with a proper definition of MDR and no XDR and PDR. However, the values of MDR recorded in the present study do not substantially deviate from earlier studies [23, 28, and 29]. The highest MDR strains were detected from E. coli (99.3%) of which 18.1% were XDR followed by K. pneumoniae (90.3%) of which 11.1% were XDR. More MDR and XDR strains in E. coli than K. pneumoniae in our study could be due to the fact that the number of E. coli strains isolated were more than K. pneumoniae. In regard to XDR and PDR, the current study is supported by the findings of previous studies [30, 31] however our finding strongly disagree with a study conducted by Ahmed Hasanin et al [32] in the prevalence of XDR strains of FGNB. In this study lower XDR E. coli (18%) and K. pneumonia (11%) were reported as compared to the previous study where higher XDR K. pneumoniae (52%), and E. coli (47%) were reported [32]. The reason for the difference in the prevalence of XDR might be due to the definition used to classify isolates into XDR and the location of the patient from (inpatient or outpatient) from where the specimen is obtained. Increased use of over-the-counter antibacterial drugs, incomplete course of therapy, and prolonged therapy for recurrent bacterial diseases are commonly practised in Ethiopia. These practices could be cited as possible factors for the high prevalence of MDR and XDR bacterial species noted in the current study.

Multidrug-resistant (MDR) strains of ESBL producing FGNB are of particular concern. The phenotypic data generated in the current study demonstrated a considerably significant prevalence of ESBL producers, where 66.8% of FGNB produced ESBL. This study is lower than other studies conducted in Ethiopia and other countries [27, 33, 34]. 78.6% overall prevalence of ESBL was reported in Addis Ababa, Ethiopia by Legese et al [27], 85.8% in Gondar, Ethiopia by Feleke Moges et al [33] and 79.3% in Tanzania by Manyahi J et al [34]. The overall prevalence of ESBL production among FGNB (66.8%) in our study is almost in line with a study conducted in Uganda [35] where 62.0% ESBL was reported. In contrast to this study, many researchers from various parts of Ethiopia was reported lower prevalence of ESBL producing FGNB. Dejene et al reported 57.7% in Addis Ababa [36], Siraj et al; 38.4% in Jimma [37], Mengistu et al; 23% in Jimma [38]. Other African countries also share a lower prevalence of ESBL than our study. In Burkina Faso (58.0%) [39], Ghana; (49.3%) [40], and in Tanzania (45.2%) [41]. Variation in the prevalence of ESBLs in different studies among clinical isolates might be due to variation in geographic areas, a period of study (ESBL rapidly changing over time), awareness in the utilization of broad-spectrum antibiotics, an infection control system, target population, sample size and method of ESBL detection.

In the current study higher prevalence of ESBL were documented in K. pneumoniae (76.4.6%) followed by E. coli (63.2%) that in line with a study conducted in Addis Ababa, Ethiopia[36]; K. pneumoniae (78.6%) and E. coli (52.2%), Jimma, Ethiopia[37]: (K. pneumoniae 70.4%, E. coli 28.2%), and Uganda[35]: (K. pneumoniae 72.7%, E. coli 58.1). However, higher ESBL prevalence was reported in E. coli than K. pneumoniae in other studies [39, 42]. Our study showed that the most common sample from which ESBL producing strains were from urine samples in lines with other studies where urine was the major source of ESBL-producers[35,43]. However, blood was reported as a major source of ESBL-producers by other researchers [36, 39]. This could probably be due to the number of strains isolated from each specimens.

Carbapenemase production in the present study was about 2% which is a much lower than prevalence rate reported in a study carried out in Gondar, Ethiopia by Feleke et al and in Addis Ababa, Ethiopia by Legese et al [27] where both of them reported a higher prevalence of 12% and 16% carbapenemase among the FGNB respectively. 2% prevalence in this study is comparable with a study conducted in Gondar, Ethiopia by Eshetie et al [23] where 2.7% K. pneumoniae were carbapenemase producer. However our finding strongly disagrees with a study done in Nigeria [44] and Tanzania [45] where the highest percentage of carbapenemase was documented (39.02%) and (35%) respectively. This difference in the prevalence of carbapenemase-producing FGNB indifferent studies might be due to extensive utilization of carbapenem antibiotics, ESBL-producing strains of K. pneumoniae, in particular, that have other resistance mechanisms to other class of antibiotics such as carbapenems might be responsible for the emergence of carbapenemase resistant strains of K. pneumoniae.

The present study demonstrated that the isolation rate of MDR strains, ESBL, and carbapenemase-producing strains of FGNB were ≥70% in an intensive care unit while the isolation of the same elements in other wards were ≤25%. Inappropriate and excessive antibiotic use, insufficient availability of infection prevention and control programs, and increased use of invasive medical devices, and invasive procedures at an intensive care units have been implicated as risk factors for the development of MDR strains and production of ESBL and carbapenemase production [46, 47]. Furthermore, more MDR, ESBL and carbapenemase-producing strains were documented in antibiotic treated subjects than that were not treated. This is given because extensive exposure of bacteria to antibacterial agents is the main factor promoting the emergence and spread of MDR, ESBL, and carbapenemase-producing bacteria.

## Conclusion and recommendations

We have noticed an increased MDR as well as ESBL among the isolated fermentative gram-negative bacilli in the study site. Very high resistance was recorded against Ampicillin, piperacillin, Sulfamethoxazole-trimethoprim and Tetracycline, hence empirical treatment with these antibiotics is not encouraged. Carbapenems resistant *Klebsiella pneumonia* was also identified in this study which is concerning the health care issue in Ethiopia. Therefore routine infection preventions strategies such as the rational use of antimicrobial agents both in Animals and humans, developing antibiotic stewardship for health facilities and implementing strong surveillance of AMR, are needed to prevent and control the spread of antimicrobial-resistant pathogens in health care settings.

### Limitations of the study

The isolates that revealed extensively and pan drug-resistant in this manuscript might be susceptible to tigecycline, colistin or fosfomycin were not tested due to lack of the antibiotics from the local market and ESBL and carbapenemase enzymes were characterized by only phenotypically. And also data used in this the study were collected from the patients referred from Addis Ababa health facilities, which don’t represent the real image of Ethiopia. Therefore, large scale study that includes all antibiotic panels, molecular epidemiology of the ESBL and carbapenemase genes and sites from different parts of the country are needed to indicate the real image of AMR in Ethiopia.

## Supporting information

S1 Raw Data Demographic data and various risk factors compiled from patients and specimens referred to EPHI during the study time period. (XLSX)
